# Thiamine-responsive megaloblastic anemia syndrome with novel compound heterozygous SLC19A2 mutations and thrombotic events: a case report

**DOI:** 10.1186/s13256-026-05960-w

**Published:** 2026-03-16

**Authors:** Francisco Xavier Jiménez, Carlos Rojas, Heidi A. Fernandez, Rossana Ruiz-Urbáez, Carlos Reyes-Silva, Jhonatan Guamán, Mauricio Carrión, Fernando Naranjo-Saltos

**Affiliations:** 1 Department of Internal Medicine, Hospital de Especialidades Eugenio Espejo, Quito, Ecuador; 2 Department of Internal Medicine, St. John’s Episcopal Hospital, Far Rockaway, NY USA; 3 Endocrinology and Diabetes Unit, Hospital de Especialidades Eugenio Espejo, Quito, Ecuador; 4 Clinical Genetics, Hospital de Especialidades Eugenio Espejo, Quito, Ecuador; 5 Department of Internal Medicine, Hospital Asdrúbal de la Torre, Cotacachi, Ecuador; 6 Department of Internal Medicine, Hospital Miguel Hilario Alcívar, Manabí, Ecuador; 7 Department of Internal Medicine, Axxis Hospital de Especialidades, Quito, Ecuador

**Keywords:** Thiamine, Megaloblastic anemia, SLC19A2 mutation, Thrombotic, TRMA, Case report

## Abstract

**Background:**

Thiamine-responsive megaloblastic anemia syndrome represents a rare autosomal recessive condition originating from mutations in the SLC19A2 gene. It is characterized by a classical triad of megaloblastic anemia, insulin-dependent diabetes mellitus, and sensorineural hearing loss. We present the case of a woman diagnosed with thiamine-responsive megaloblastic anemia, with no history of consanguinity, in whom genetic testing revealed novel SLC19A2 mutations and an unreported clinical manifestation.

**Case Presentation:**

This case describes an 18-year-old mestizo female patient who presented with a medical history of diabetes starting in infancy, bilateral sensorineural hearing loss, and megaloblastic anemia. The diagnosis of thiamine-responsive megaloblastic anemia was confirmed by genetic testing, which detected compound heterozygous mutations in the SLC19A2 gene that included a pathogenic frameshift mutation (c.620_624dup; p.Pro209Phefs*21) and a missense variant (c.170 T > C; p.Leu57Pro). The patient also experienced thrombotic events, including deep vein and mesenteric thrombosis, previously unreported findings in thiamine-responsive megaloblastic anemia. High-dose thiamine treatment resulted in improved hematologic and glycemic control.

**Conclusion:**

This case broadens the genetic and clinical spectrum of thiamine-responsive megaloblastic anemia, highlighting the importance of genetic testing in young patients with the classic triad and showing that early thiamine therapy can markedly improve outcomes.

## Background

Thiamine-responsive megaloblastic anemia (TRMA) syndrome was first reported by Rogers *et al*. in 1969. It is a rare autosomal recessive disease characterized by the classic triad of megaloblastic anemia, insulin-dependent diabetes mellitus, and sensorineural hearing loss. TRMA may also present with additional clinical manifestations such as cardiovascular malformations, arrhythmias, stroke, seizures and visual disturbances. It typically manifests during infancy or adolescence and frequently affects families with a history of consanguinity [1, 2].

To date, 183 patients with TRMA from 138 families have been identified, with genetic testing confirming the diagnosis in 155 cases. Although TRMA has been reported worldwide, the majority of cases originate from the Middle East (37.7%) and South Asia (21.9%) [3].

TRMA is caused by mutations in the SLC19A2 gene, which encodes thiamine transporter 1 (THTR-1), a high-affinity thiamine transporter located on chromosome 1q23.3. THTR-1 is a high-affinity carrier protein essential for cellular uptake of thiamine in tissues such as the bone marrow, pancreatic β-cells, and cochlear cells, which would explain the classic triad of symptoms [1, 4].

TRMA responds clinically to high-dose thiamine. In most cases, this therapy corrects the anemia, which would otherwise be transfusion-dependent, while also improving glycemic control [1]. Prompt diagnosis is crucial, given the potential for clinical improvement with early treatment.

This case highlights an 18-year-old female patient with genetically confirmed TRMA and novel thrombotic events, expanding the clinical spectrum and reinforcing the importance of genetic testing in diagnosis.

### Case presentation

**Fig. 1:**
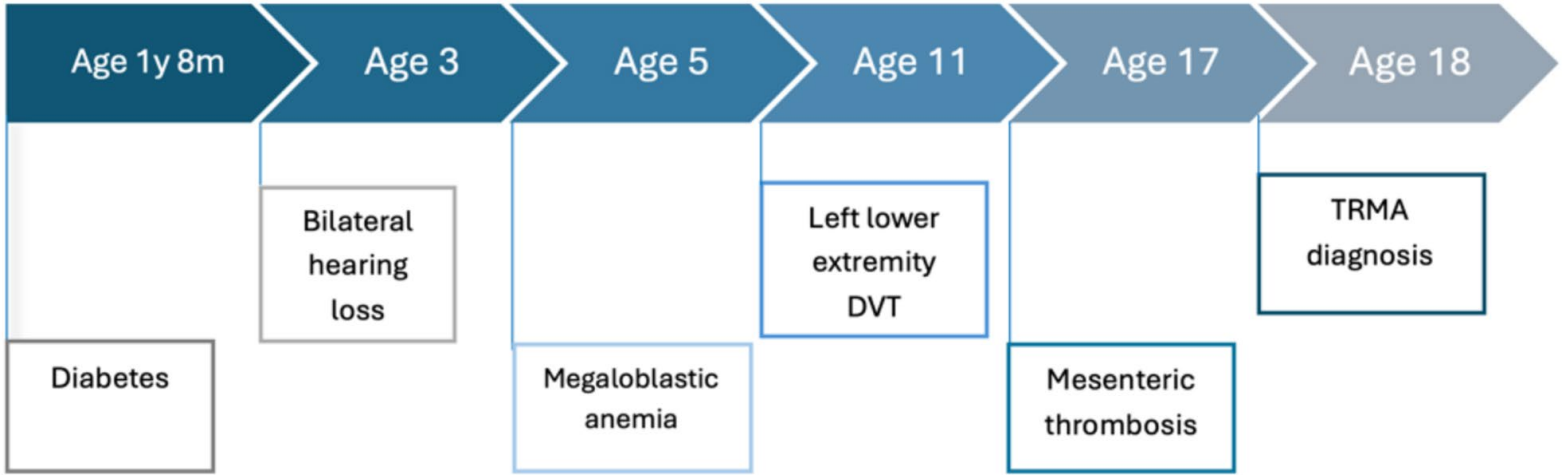
Timeline of clinical events in a patient with thiamine-responsive megaloblastic anemia syndrome. Created by the authors based on the patient’s clinical course

We report the case of an 18-year-old mestizo woman who presented with dyspnea on exertion, fatigue, and poor appetite. Her medical history was notable for diabetes diagnosed when she was 1 year and 8 months old. The patient experienced persistent suboptimal glycemic control despite receiving high-dose insulin therapy. At age 3 years she was diagnosed with bilateral sensorineural hearing loss that led to an estimated 65% hearing impairment. By age 5 years, she developed megaloblastic anemia, which was poorly responsive to treatment and required periodic blood transfusions. At age 11 years she experienced two episodes of deep vein thrombosis (DVT) in her left lower extremity followed by mesenteric thrombosis at age 17 years. Extensive evaluation for hypercoagulable states yielded negative results. She is currently on anticoagulation therapy (Fig. 1).

Family history is notable for type 2 diabetes mellitus and Parkinson’s disease (maternal grandfather). There were no reported significant prenatal or perinatal events; she was born to Ecuadorian parents and has no known history of consanguinity.

At presentation, she reported dyspnea on exertion, fatigue, and hyporexia. On physical examination, she exhibited generalized pallor and had confirmed bilateral sensorineural hearing loss. Ophthalmologic and cardiovascular examinations were unremarkable.

### Laboratory test findings

Laboratory testing revealed severe macrocytic anemia, with a hemoglobin of 5.6 g/dL (12.0–15.5 g/dL), hematocrit of 16.9% (36–46%), mean corpuscular volume of 103.7 fL (80–100 fL), and a mean corpuscular hemoglobin concentration of 34.4 pg (27–33 pg). Red cell distribution width (RDW) was elevated at 28.4% (11.5–14.5%) and the platelet count was slightly reduced to 114,000/µL (150,000–450,000/µL). Coagulation studies were within normal limits, with a prothrombin time of 14 seconds (11–14 seconds), an international normalized ratio of 1.24 (0.8–1.2), and a partial thromboplastin time of 32.1 seconds (25–35 seconds). The metabolic panel showed a creatinine level of 0.58 mg/dL (normal: 0.6–1.3 mg/dL), hyperglycemia with glucose level of 298 mg/dL (normal fasting: 70–100 mg/dL), and a hemoglobin A1c (HbA1c) of 8.8% (normal: 4–5.6%). Ferritin, vitamin B12, and folic acid were within normal limits.

We considered a diagnosis of TRMA syndrome in our patient on the basis of the classical triad of megaloblastic anemia, sensorineural hearing loss, and early onset diabetes requiring insulin therapy, all of which manifested during early childhood.

### Genetic testing

Genetic analysis was performed using a panel-based next-generation sequencing approach that revealed two mutations in the SLC19A2 gene: a pathogenic variant, c.620_62 4dup (p.Pro209Phefs*21), and a likely pathogenic mutation, c.170 T > C (p.Leu57Pro).

Parental genetic testing confirmed the heterozygous carriage of the respective variants: the c.620_624dup (p.Pro209Phefs*21) mutation was identified in the father, and the c.170 T > C (p.Leu57Pro) mutation was identified in the mother (Table 1).

**Table 1:**
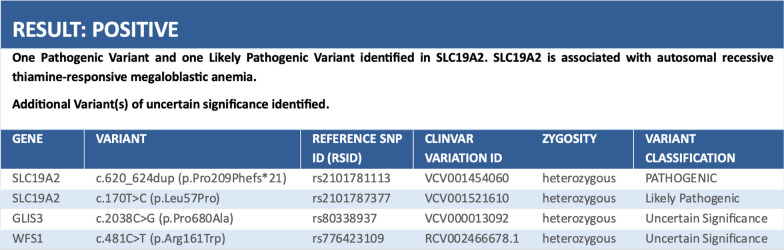
Genetic testing results from the molecular diagnostics laboratory at Hospital Eugenio Espejo, identifying compound heterozygous variants in SLC19A2 consistent with TRMA syndrome

Data obtained from the patient’s clinical record.

### Treatment and outcome

Following confirmation of the diagnosis of TRMA, we started the patient on intravenous high-dose thiamine (100 mg) daily which was changed to oral prior to discharge. During her stay she received one unit of packed red blood cells, which increased her hemoglobin to 7.2 g/dL, and further improved to 8.1 g/dL by the time of discharge. At her outpatient follow-up, she showed significant hematologic improvement, with hemoglobin levels increasing to 8.1 g/dl at 1 month and 14 g/dL with Mean corpucular volume (MCV) of 81 fl, mean corpuscular hemoglobin of 25 pg and RDW of 22% at 8 months and no further need for blood transfusions.

Glycemic control also improved progressively, with a mild but consistent reduction in daily insulin requirements. Her HbA1c decreased to 7.4% after 8 months of daily oral thiamine, which was well tolerated without any adverse events. Adherence was confirmed through patient self-report and clinical response. No additional thrombotic events occurred, but there was no reported improvement in the patient’s hearing loss.

## Discussion

TRMA syndrome (Online Mendelian Inheritance in Man #249,270) is a rare autosomal recessive multisystem disorder characterized by the classical triad of megaloblastic anemia, progressive sensorineural hearing loss, and diabetes. It results from biallelic pathogenic variants in the SLC19A2 gene, which encodes thiamine transporter 1 (THTR-1), a membrane protein essential for cellular thiamine uptake that shields its positive charge from repulsive membrane forces. THTR1 facilitates thiamine transport, particularly in the bone marrow, pancreatic β-cells, and cochlear cells [4, 5].

THTR-1 deficiency impairs intracellular thiamine transport in β-islet cells and disrupts aerobic metabolism. As a result, β-cell loss and decreased insulin secretion occur, leading to metabolic dysfunction [6, 7]. Megaloblastic anemia in TRMA is attributed to reduced thiamine-dependent transketolase activity, which limits nucleotide synthesis and causes ineffective erythropoiesis with enlarged, undivided red cell precursors [2]. Neurologic and cardiovascular abnormalities, including sensorineural hearing loss, may be linked to underlying mitochondrial dysfunction [5].

In our case, a monogenic diabetes panel revealed novel compound heterozygous mutations in the SLC19A2 gene. A pathogenic frameshift mutation, c.620_624dup (p.Pro209Phefs*21), and a likely pathogenic missense variant, c.170 T > C (p.Leu57Pro) were identified. The frameshift variant introduces a premature stop codon and has not been previously reported in population databases or in association with SLC19A2-related conditions. The second variant replaces leucine with proline at codon 57 of the SLC19A2 protein, which affects the highly conserved leucine residue. This missense variant is also not present in population databases and is predicted to disrupt protein function due to physicochemical differences between leucine and proline. Leucine is flexible and hydrophobic, allowing it to fit comfortably into α-helices and β-sheets and help maintain proper protein folding. In contrast, proline has a rigid cyclic structure that introduces a kink in the in the affected segment of the protein chain, disrupting local secondary structures. This substitution is therefore likely to alter the folding and stability of the thiamine transporter, impairing its function [8]. Parental genetic testing confirmed the inheritance of one mutation from each parent, which was consistent with autosomal recessive transmission.

Previous studies have documented different mutations in the SLC19A2 gene. In a cohort of 111 families from 31 countries, 74 mutations were identified: 71.6% were homozygous, 20.3% were compound heterozygous, and 8.1% were both [4]. The most commonly reported variants were c.241_242insA (ten families), c.196G > T (seven families), c.697C > T (seven families), and c.515G > C (five families) [4].

Although consanguinity was reported in 61.8% of 157 patients in a previous review, it was not present in our case. The syndrome shows no sex predilection. A review of 23 cases from two registries found that 52% of the patients were male. Geographically, TRMA shows a higher prevalence in the Middle East (37.7%) and South Asia (21.9%), according to a review of 183 patients from 88 publications [4, 9]. To our knowledge, no other cases have been previously reported in Ecuador. Although our patient exhibited typical features from infancy, diagnosis was delayed. The average age of symptom onset is approximately 10 months, and time to diagnosis is approximately 8 years [10, 11].

Clinical diagnosis of TRMA relies on the presence any of the features of the classic triad, family history, and partial symptom resolution with thiamine supplementation. Definitive diagnosis is established through genetic testing, which would reveal mutations in the SLC19A2 gene [12]. In this case, the presence of pathogenic variants in SLC19A2 also supports TRMA as the underlying etiology of her insulin dependence, rather than classic autoimmune diabetes.

In a systematic review of 151 patients, megaloblastic anemia was observed in 95.4%, diabetes in 92.7%, and hearing loss in 92.7%, with 84.1% showing the full triad. Other features included neurologic deficits and developmental delays (37.5%), visual defects (25%), cardiovascular malformations (15.6%), thrombocytopenia (12.5%), and short stature (9.4%) [13]. Our patient exhibited the classic triad with one of these additional manifestations. Ophthalmologic and cardiovascular evaluations were normal.

Notably, our patient had a history of lower extremity DVT and mesenteric vein thrombosis, findings not previously described in TRMA. Extensive evaluation for hypercoagulable states were negative. Although thrombotic events in TRMA are uncommon and these specific sites have not been previously reported, some studies have described comparable thrombotic complications. One study reported a patient with TRMA who developed unprovoked pulmonary embolism and portal vein thrombosis, suggesting a possible link to the variant in TPK1:NC_000007.13 (TPK1_v002):c.613 + 39 T > C [14]. Xi *et al*. and Villa *et al*. each described a patient with TRMA who suffered a stroke [8, 15]. To our knowledge, this is the first reported case of TRMA presenting with both DVT and mesenteric vein thrombosis, and adds to this emerging evidence by describing them as possible novel complications. Although the precise mechanism linking SLC19A2 mutations to thrombosis remains speculative, these cases collectively suggest a potential association between TRMA and an increased risk of thrombotic events. Further investigation into the potential genetic mechanisms linking the two is needed, as no clear genetic basis has been established to date.

Following the diagnosis, our patient was started on daily high-dose thiamine supplementation. She achieved a satisfactory hematologic response that resulted in hemoglobin levels returning to normal and no longer required transfusions. Glycemic control improved over time, with a reduction in insulin requirements and improvement of HbA1c. These findings are consistent with previously reported outcomes, such as a cohort of 140 patients treated with thiamine, where 97.1% showed hematologic improvement and 69.9% reported reduced or eliminated insulin requirements [13]. However, hearing improvement is rare and has been observed in only two reported cases.

Some aspects of this report are limited by available resources and testing. In many parts of our country, access to advanced tests such as autoantibody panels for type 1 diabetes or comprehensive genetic studies is limited by financial, cultural, and institutional barriers. In this case, a monogenic diabetes panel was available and revealed only SLC19A2 mutations, which explains her diabetes phenotype. Future reports may benefit from including such testing to further clarify the differential diagnosis. Another limitation is that thiamine levels were not measured due to unavailability at our institution. However, this did not affect the diagnosis as thiamine levels are often normal in TRMA, and confirmation relies primarily on genetic testing and clinical response to therapy. However, such resources are not routinely available in all hospitals, which may explain why diagnoses such as TRMA are often delayed.

Managing TRMA can be challenging not only medically, but also financially and emotionally for patients and their families. Genetic testing can be expensive, especially in settings where patients must pay out-of-pocket as many hospitals do not have access to them. These costs, combined with frequent hospital visits and the difficulty of facing illness at a young age, can place a significant psychological burden on patients and their families. Expanding access to genetic and antibody testing could help clinicians distinguish TRMA from more common conditions, such as type 1 diabetes, and allow earlier initiation of effective treatment with thiamine.

## Conclusion

This case highlights the importance of early recognition and genetic confirmation of TRMA syndrome, particularly in young patients with features of the characteristic triad. The identification of novel compound heterozygous mutations in SLC19A2 confirmed the diagnosis in our patient, who had no history of consanguinity. Of particular interest was the occurrence of thrombotic events, which have not been previously associated with TRMA. A genomic basis for this finding has yet to be found and may represent a novel clinical feature requiring further study. Timely initiation of thiamine therapy led to marked clinical improvement, emphasizing the need for increased awareness of this rare but treatable condition.

## Data Availability

Not applicable.

## Abbreviations

TRMA: Thiamine-responsive megaloblastic anemia
DVT: Deep vein thrombosis
HbA1c: Hemoglobin A1c
THTR-1: Thiamine transporter 1
